# Special Hospitals: VI. Miscellaneous. Cost of Management


**Published:** 1893-10-14

**Authors:** 


### The Institutional Workshop.

### HOSPITAL ADMINISTRATION.

### VI.—SPECIAL HOSPITALS: MISCELLANEOUS. COST OF MANAGEMENT.*

#### Miscellaneous Hospitals.

Besides the various groups of special hospitals which we have dealt with in previous articles there remains 20 others which it is difficult to classify. We have therefore grouped them all as miscellaneous, but have divided them into two lists, viz.: (a) those with 100 beds and upwards; and (b) those with 50 beds and under. This is anything but an ideal arrangement, but it is the one which seems most instructive, and so we adopt it.

It will be seen from the following table that there are five miscellaneous hospitals, containing 100 beds and upwards:—

compared with 5·538 per cent. in 1891, the outlay under this head is so small as to warrant the committee in expending more money upon salaries at their discretion.

We are glad to be able to record a reduction in the percentage of cost of administration at the Cancer Hospital, Brompton, from 18·033 in 1891 to 16·623 per cent. in 1892. We hope this reduction will encourage the committee to persevere, and then no doubt the cost of administration will be reduced to 15 per cent., thus bringing the Cancer Hospital within the prescribed limits, which entitle an institution of this type to claim to be classed among the efficiently managed. This further reduction is foreshadowed by the fact that the percentage of cost of administration to the total ordinary expenditure in 1892 was only 14·254 per cent.

(a) Large Miscellaneous Metropolitan Hospitals.

| Name of Hospital. | Beds for Occupation. | Average Number Occupied. | Total In-patients Admitted. | Total Out-patients Admitted. | Percentage of cost of Administration to that of Maintenance. | Percentage of Administration to Total Expenditure. |
|---|---|---|---|---|---|---|
| London Fever ... ... ... ... ... ... | 200 | 98 | 748 | — | 22·724 | 18·511 |
| National for the Paralysed and Epileptic ... | 170 | 157 | 828 | 4,281 | 11 882 | 10·620 |
| Female Lock ... ... ... ... ... ... | 135 | 97 | 706 | — | 16·397 | 14·087 |
| Cancer, Brompton ... ... ... ... ... | 105 | 75 | 811 | 1,289 | 16·623 | 14·254 |
| London Temperance ... ... ... ... ... | 120 | 70 | 859 | 8,190 | 5·676 | 5·371 |

None of the hospitals included in the table, except the first, present any special features which warrant an expenditure upon management greater than that laid down for general hospitals, viz., 15 per cent. It is satisfactory to be able to report that the percentage of the cost of administration to the total expenditure, in every case to which the limit applies, was less than 15 per cent. during the year 1892.

The exception is the London Fever Hospital, which has to be maintained continuously in an efficient condition, ready to admit patients although few fever cases may prevail in London, and so the annual expenditure upon maintenance in a given year may appear altogether out of proportion to that upon administration. The outlay on administration at all fever hospitals is necessarily large and continuous, though the expenditure upon maintenance may be comparatively small during any given year. The justice of these contentions is confirmed by the fact that, whereas in 1891 the London Fever Hospital had only 57 out of 200 beds continuously occupied, and the total in-patients amounted to 515, the percentage of cost of administration to that of maintenance was 25·13 per cent. During the year 1892 98 beds were constantly occupied, 748 in-patients were treated, and the percentage of cost of administration fell to 22·724 per cent. This is satisfactory in every way, especially as the percentage of administration to total ordinary expenditure amounted to only 18·551 per cent.

The London Temperance Hospital has always been economically administered, and although the percentage of cost of administration to that of maintenance shows a slight increase, viz., 5·676 per cent. in 1892, as

* Previous articles of this series have appeared in The Hospital, page 333 for August 13th; page 349 for August 26th; page 381 for September 9th; page 413 for September 23rd; and page 429 for September 30th.

The National Hospital for the Paralysed and Epileptic is one of the most indispensable, as it is one of the best managed of metropolitan institutions. It was highly credible to the authorities, having regard to the special difficulties they have to face in raising income, that the expenditure upon management in 1891 should have been less than 14 per cent. They have, however, improved their position in 1892 by expending only 11·882 per cent. on administration compared with maintenance. Mr. Burford Rawlings, the able Secretary of the National Hospital, is to be warmly congratulated upon these figures, and we should be glad to hear that his committee having taken them into their most favourable consideration, have seen their way to reward him adequately in consequence. As we urged last year, the most ardent advocate of special hospitals, who regards lavish expenditure upon management as a detail, must be forced to admit in all fairness that, if Mr. Rawlings is able to maintain the high state of efficiency which prevails at the National Hospital by an expenditure of less than 12 per cent. upon management, there is no reason why every other special hospital should not reduce its expenditure so as to bring it into line with the best administered of this class of medical charities The results achieved at the National Hospital prove to demonstration that 15 per cent. is the maximum sum which ought to be spent upon management by any of the hospitals included in the above table, with the exception of the London Fever Hospital.

#### (b) Smaller Miscellaneous Metropolitan Hospitals.

The following table, containing the names of 15 smaller special hospitals concludes our list, and shows what a miscellaneous variety of hospitals London possesses. Only one of the 15 hospitals—the National

Orthopædic, with 63 beds—contains more than 50 beds, the number in this institution having been increased by 28 during the year 1892.

The Throat Hospital, Golden Square, takes the blue ribbon with the lowest percentage of cost of administration, viz., 8·392 per cent. Next in order comes the

Smaller Miscellaneous Metropolitan Hospitals.

| Name of Hospital. | Beds for Occupation. | Average Number Occupied. | Total In-patients Admitted. | Total Out-patients Admitted. | Percentage of cost of Administration to that of Maintenance. | Percentage of Administration to Total Expenditure. |
|---|---|---|---|---|---|---|
| Royal Orthopædic ... ... | 50 | 47 | 171 | 1,071 | 19·932 | 16·619 |
| National Orthopædic ... | 63 | 57 | 216 | 660 | 19·843 | 16·557 |
| St. Mark's, for Fistula ... | 34 | 20 | 296 | 731 | 22·607 | 18·438 |
| City Orthopædic ... ... | 27 | 19 | 161 | 1,950 | 25·432 | 20·270 |
| St. Peter's, for Stone ... | 26 | 15 | 367 | 4,420 | 20·593 | 17·077 |
| Throat Hospital, Golden Sq. | 25 | 22 | 633 | 7,941 | 8·392 | 7·743 |
| National Heart and Paralysis | 25 | 23 | 108 | 1,762 | 23·130 | 18·789 |
| Epilepsy and Paralysis, Regent's Park ... ... | 25 | 17 | 97 | 731 | 22·640 | 18·460 |
| Male Lock ... ... ... | 20 | 14 | 274 | 3,863 | 10·291 | 9·320 |
| Central London Throat and Ear ... ... ... | 16 | 13 | 288 | 6,250 | 11·076 | 10·000 |
| Gordon, for Fistula ... ... | 11 | 9 | 134 | 526 | 12·744 | 11·303 |
| Stamford Street Skin ... | 10 | 3 | 17 | 5,099 | 15·968 | 13·769 |
| Royal Ear ... ... ... | 9 | 6 | 179 | 2,596 | 18·074 | 15·307 |
| London Dental ... ... | — | — | — | 50,480 | 24·000 | 19·692 |
| National Dental ... ... | — | — | — | 23,847 | 24·899 | 19·916 |

With every desire to do justice in the most liberal spirit to the smallest special hospital, we are bound to repeat that, in our judgment, in none of these institutions ought the percentage of the cost of administration to that of maintenance to exceed 20 per cent. It is gratifying to find that the percentage of administration to the total ordinary expenditure is under 20 per cent. in the case of every institution, except the City Orthopædic Hospital, where it amounts to 20¼ per cent. The percentage of cost of administration to that of maintenance in 1892 compared with 1891 shows the following reductions:—

| | Per Cent Reduction. |
|---|---|
| Royal Orthopædic Hospital ... ... ... | 2 |
| National Orthopædic Hospital ... ... ... | 1 |
| St. Mark's Hospital... ... ... ... ... | ¾ |
| City Orthopædic Hospital... ... ... ... | 1¼ |
| Throat Hospital ... ... ... ... ... | 2½ |
| National Heart ... ... ... ... ... | 1 |
| Epilepsy and Paralysis Hospital... ... ... | ½ |
| Male Lock ... ... ... ... ... ... | ¾ |
| Central London Throat Hospital, nearly. ... | 1 |
| Gordon Hospital, nearly ... ... ... ... | 2¼ |
| Stamford Street Skin Hospital ... ... ... | ⅔ |
| The London Dental... ... ... ... ... | ½ |

This considerable and practically uniform lowering of the cost of administration at the smaller miscellaneous metropolitan hospitals is a remarkable fact and a most encouraging one also. We congratulate the authorities upon the wisdom which has dictated the attempt to curtail the expenditure, and the success which has attended the effort. Nothing more creditable to the small special hospitals has probably happened in the whole course of their history.

On the other hand, we are sorry to see that the percentage of cost of administration to maintenance at the Royal Ear Hospital has increased from 14·531 in 1891 to 18·074 per cent. in 1892. We have no doubt that the unenviable distinction thus obtained by the Royal Ear Hospital will cause the management to seriously consider their position, and the effect which this increase must have upon their revenue if it is not promptly remedied.

Male Lock with 10·291 per cent., the Central Throat and Ear with 11·076 per cent., and the Gordon for Fistula with 12·704 per cent. All these institutions deserve praise for economy—a fact which we have no doubt will be duly appreciated by the giving public when they come to distribute their cheques amongst the medical charities at the end of the year.

In these circumstances the expenditure upon management of over 25 per cent. by the City Orthopædic Hospital; of over 23 per cent. by the National Heart and Paralysis Hospital; and of 22·6 per cent. by St. Mark's Hospital and the Hospital for Epilepsy and Paralysis is remarkable, although we are glad to find a reduction as compared with previous years, pointing to a steady continuance in the effort to bring these institutions into line by reducing the cost of management to a maximum of 20 per cent. The two dental hospitals, with an expenditure on management in round numbers of 24 and 25 per cent., leave much to be desired. We should further be glad to have some explanation of the causes which produce an expenditure on management of only 10·291 per cent. at the Male Lock, whilst the cost at the Female Lock, which contains 135 beds, amounts to over 16 per cent.

It has been our practice to refer our readers to previous articles dealing with the cost of management of all the metropolitan hospitals, because they are published with the view of helping all who support our hospitals to encourage economy in administration. The series of special articles we have published on the metropolitan hospitals ought to prove serviceable by enabling all who desire to give wisely to secure that the greater proportion of their gifts shall go to relieve the sufferers whom they desire to benefit. The precise and accurate figures, with the explanations given, contained in the articles which we have published, should be carefully noted by the giving public when they next distribute their gifts among the hospitals.

